# Exploring Hypertension: The Role of AT1 Receptors, Sartans, and Lipid Bilayers

**DOI:** 10.1021/acsomega.4c06351

**Published:** 2024-11-01

**Authors:** Nikitas Georgiou, Eleni Chontzopoulou, Efthymios Alexandros Routsi, Irene Georgia Stavrakaki, Errikos Petsas, Nikoletta Zoupanou, Margarita Georgia Kakava, Demeter Tzeli, Thomas Mavromoustakos, Sofia Kiriakidi

**Affiliations:** † Laboratory of Organic Chemistry, Department of Chemistry, National and Kapodistrian University of Athens, Panepistimiopolis Zografou, 15771 Athens, Greece; ‡ Industrial Chemistry Laboratory, Department of Chemistry, National and Kapodistrian University of Athens, 10679 Athens, Greece; § Laboratory of Organic Chemistry and Biochemistry, Department of Chemistry, University of Patras, 26504 Patras, Greece; ∥ Laboratory of Physical Chemistry, Department of Chemistry, National and Kapodistrian University of Athens, Panepistimiopolis Zografou, 15771 Athens, Greece; ⊥ Theoretical and Physical Chemistry Institute, National Hellenic Research Foundation, 48 Vassileos Constantinou Avenue, 11635 Athens, Greece; # Departamento de Quimica Orgánica, Facultade de Quimica, Universidade de Vigo, 36310 Vigo, Spain

## Abstract

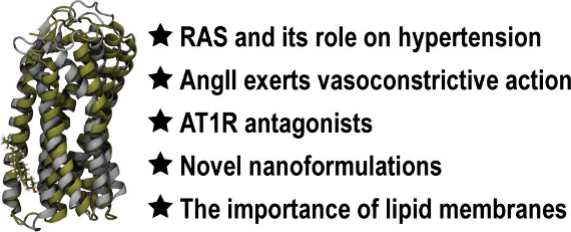

The rational design of AT1 receptor antagonists represents a pivotal approach in the development of therapeutic agents targeting cardiovascular pathophysiology. Sartans, a class of compounds engineered to inhibit the binding and activation of Angiotensin II on the AT1 receptor, have demonstrated significant clinical efficacy. This review explores the multifaceted role of sartans in mitigating hypertension and related complications. We highlight the integration of crystallography, computational simulations, and NMR spectroscopy to elucidate sartan-AT1 receptor interactions, providing a foundation for the next-generation antagonist design. The review also delves into the challenges posed by the high lipophilicity and suboptimal bioavailability of sartans, emphasizing advancements in nanotechnology and novel drug delivery systems. Additionally, we discuss the impact of lipid bilayers on the AT1 receptor conformation and drug binding, underscoring the importance of the lipidic environment in receptor-drug interactions. We suggest that optimizing drug design to account for these factors could enhance the therapeutic potential of AT1 receptor antagonists, paving the way for improved cardiovascular health outcomes.

## 1 Introduction

One of the most challenging issues worldwide is to ensure the quality of life and reduce, or in the best-case scenario eradicate, the risk of death due to life-threatening diseases. Hypertension belongs to the latter, and up to 7.8 million premature deaths are estimated due to cardiovascular disease by 2025.^1^ Hypertension is the major risk factor for cardiovascular diseases, a chronic illness with a high mortality rate.

**Figure 1:**
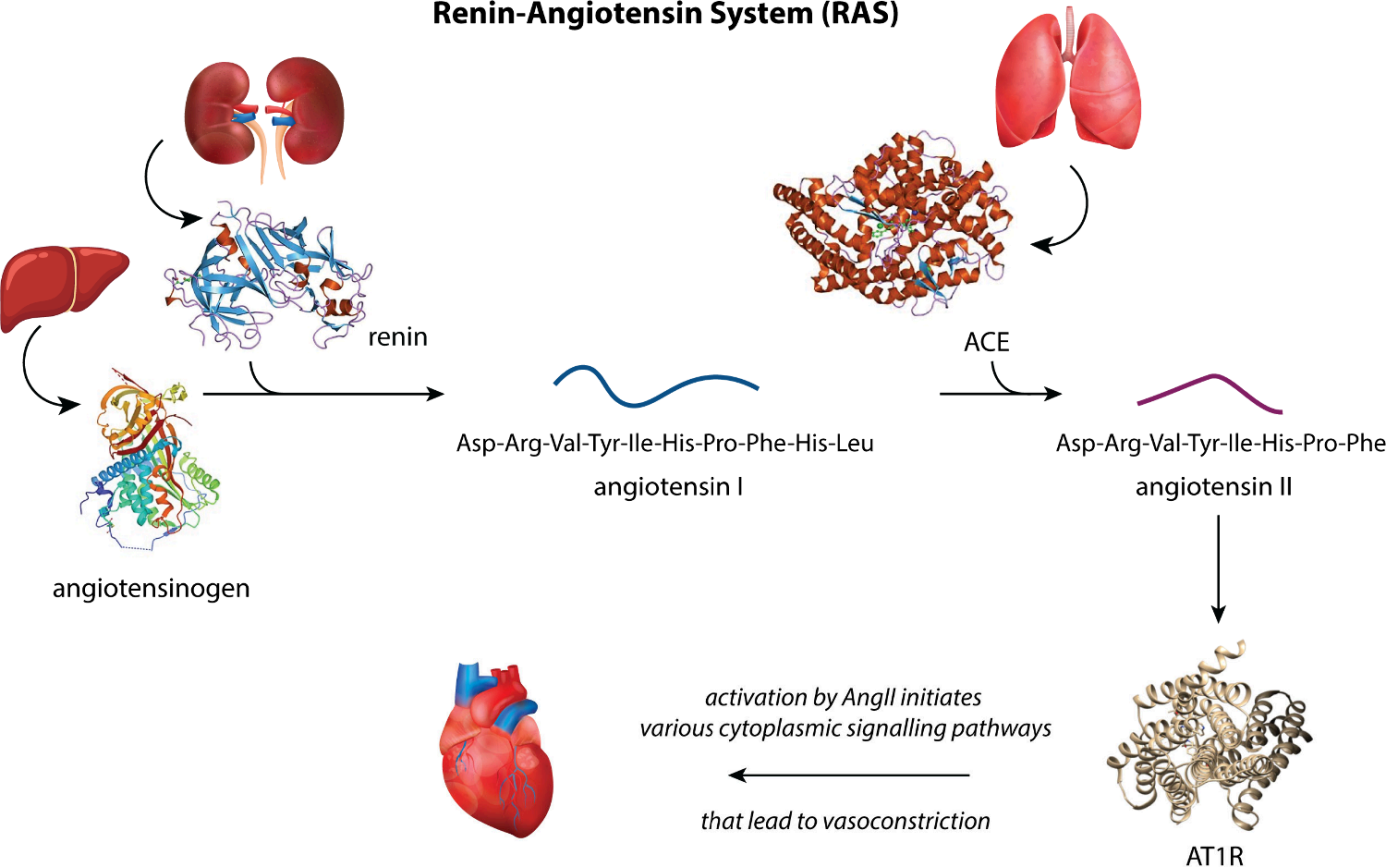
A schematic representation of the RAS system consisting of the enzyme renin and its substrate angiotensinogen, the oligopeptide angiotensin I, the ACE which converts angiotensin I to angiotensin II and, finally, the GPCR AT1R to which angiotensin II physiologically binds. The images of liver, lungs, kidneys and heart were downloaded under the Free License of www.vecteezy.com. The images of renin, angiotensinogen, ACE and AT1R are public domain from wikipedia.org.

**Scheme 1:**
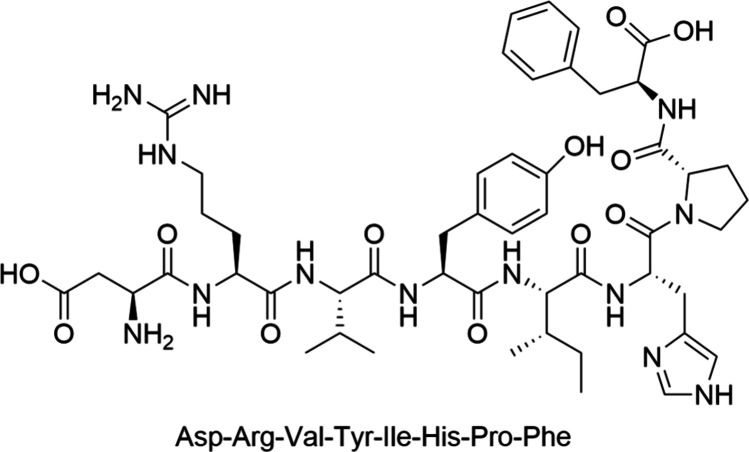
Sequence and 2D Structure of AngII

Hypertension^2−4^ is defined as abnormally high blood pressure (hyper in Greek means excessive) which, if it remains high for extended periods, can lead to damage of the arterial wall. A sedentary lifestyle, daily consumption of ultra-processed foods, and smoking are the major factors that lead to high blood pressure. There are various pharmaceutical compounds used to reduce the risk of hypertension, depending on the target within the Renin-Angiotensin System (RAS). The RAS is a hormone system that regulates blood pressure, fluid balance, and vascular resistance in the human body. It is activated in response to various stimuli, such as low blood pressure, decreased sodium chloride in the kidney tubules, or sympathetic nervous system activation. It begins with the secretion of renin, an enzyme produced by the juxtaglomerular cells of the kidneys.^5^ Renin catalyzes the conversion of angiotensinogen, a heterogeneous glycoprotein produced by the liver,^6^ into Angiotensin I (AngI). This conversion is highly regulated from the degree of the substrate’s glycosylation.^7^ AngI is relatively inactive but is quickly converted into Angiotensin II (AngII) by the action of the angiotensin-converting enzyme (ACE), primarily in the lungs.^8^ AngII, presented in Scheme 1, is a highly effective vasoconstrictor that acts on the Angiotensin II Type 1 Receptor (AT1R) and induces the narrowing of blood vessels, thereby elevating blood pressure. Moreover, it promotes the secretion of aldosterone from the adrenal cortex. Aldosterone acts on the renal tubules to enhance sodium reabsorption, which leads to increased water reabsorption into the bloodstream, while simultaneously facilitating potassium excretion to maintain electrolyte balance. The resulting increase in extracellular fluid volume further contributes to the elevation of blood pressure.^9^ Due to the role of aldosterone in regulating blood pressure, RAS is often referred to as RAAS (renin-angiotensin-aldosterone system). The main components of the RAS are presented in Figure 1.

The AT1R is a G-protein-coupled receptor (GPCR) embedded in membrane bilayers, and it is mostly expressed in the sarcolemma membrane. Previous works conducted by our group^10−16^ and others^17−20^ highlight the importance of the lipidic environment on the receptor’s conformation and action. Thus, although formally not a part of the RAS, we highlight the role of the lipidic membranes on the regulatory action of this hormonal system. AngII binds to the AT1R and activates it via stacking interactions between Phe8 (AngII)/His256(AT1R) and Tyr4(AngII)/Asn111(AT1R) that results in conformational changes in the transmembrane helices of the receptor. These changes induce a variety of signaling pathways in the cytoplasmic region, which include interaction with trimeric G-proteins and the activation of a variety of intracellular protein kinases, including the mitogen-activated protein kinase (MAPK) family. The receptor’s internalization is induced by another family of proteins, the β-arrestins,^21^ a protein family which in addition to AT1R desensitization and endocytosis, initiates additional signaling cascades. When AT1R is overactivated by AngII, or when its biodegradation is impaired (e.g., due to lack of specific glycosylation^22^), pathological conditions that lead to hypertension arise. A class of drugs that acts on AT1R to block the harmful effects of AngII, is that of the AT1R antagonists, which are polydynamic molecules with diverse biological effects. In this review article we will focus on their action on RAS and more specifically their antagonism to the peptide hormone AngII. Other strategies to treat hypertension also exist, such as beta blockers, diuretics, or ACE inhibitors.

**Scheme 2:**
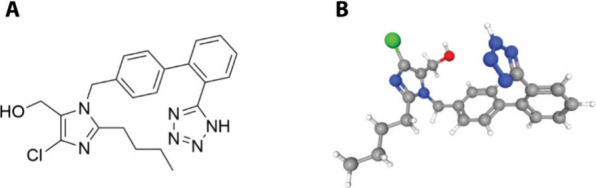
Structure of Losartan in (a) 2D and (b) 3D View

Losartan (presented in Scheme 2) serves as a prototype within the category of AT1 antagonists, acting with selectivity on AT1R to inhibit the detrimental effects induced by AngII under pathological conditions. Its development was based on rational drug design methodologies, in an attempt to achieve a nonpeptidic, peptidomimetic analogue that can act on AT1R. Several optimization rounds led to the conclusion that the best analogue for the acidic groups of Asp1 and Tyr4 would be a tetrazole ring. Moreover, the replacement of the two aromatic rings of AngII by the biphenyl group led to improved bioavailability. An aliphatic chain, like the one present in Ile5 was also necessary for an effective analogue. Finally, the imidazole of His6 was deemed important and was kept in the losartan molecule.^23,24^ After years of development and clinical studies, losartan got the FDA approval in 1995.^24^ It is administered orally as an antihypertensive drug in the form of a potassium salt.^25,26^ Recently, losartan has been proposed as a promising treatment for COVID-19.^27^ Additionally, it is utilized to decrease the risk of stroke in patients with left ventricular hypertrophy and in the management of diabetic nephropathy. It has also been tested for use in myocardial infarction treatment. The maximum hypotensive effect typically becomes evident within 3–6 weeks of initiating treatment.

Losartan has a molecular weight below 500 g/mol, with fewer than 5 hydrogen bond donors, less than 10 hydrogen bond acceptors, and a lipophilicity of under five. Consequently, it adheres to Lipinski’s Rules of Five.^28^ Moreover, it meets Veber’s Rule^29^ as it has fewer than seven rotatable bonds. Its water solubility is notably low, resulting in poor bioavailability. Moreover, losartan has been shown to have antihypertensive activity,^30^ protection against diabetic nephropathy,^31−41^ and heart failure,^42−46^ prevention of stroke,^47,48^ migraine prophylaxis,^49,50^ anti-inflammatory effect,^51−66^ antifibrotic effect,^67−69^ and anticancer activity.^70,71^ Due to its mode of action, losartan avoids the side effects associated with calcium antagonists and has consequently served as the basis for an entire family of analogues known as “the sartans”.^23^ This family consists of 8 commercially available sartans, namely losartan, candesartan, valsartan, telmisartan, irbesartan, eprosartan, azilsartan, and olmesartan (presented in Table 1). The widespread global use of sartans has prompted a recent review by Ladhari et al.^72^ on the potential environmental risks of their degradation products or their accumulation in drink and waste waters.

**Table 1:**
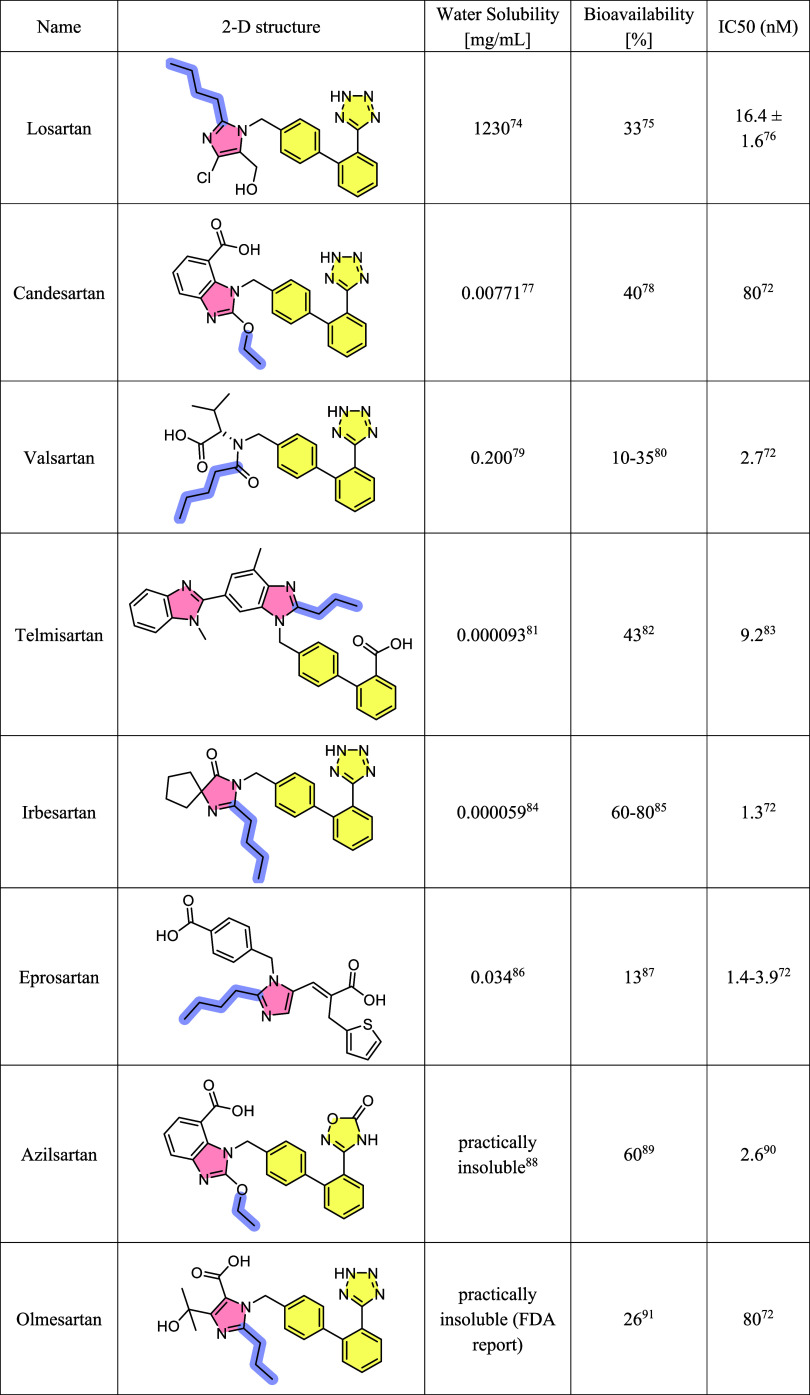
Eight Commercially Available Sartans Are Presented along with Their Water Solubility, Their Bioavailability, and Their IC50 Values against the AT1R^a^^74−91^

a Their 2D structures are highlighted in order to pinpoint their common structural characteristics that consist of a biphenyl (tetrazole), highlighted in yellow, an imidazole, in pink, and an alkyl chain, in purple.

All eight sartans share some common characteristics in their scaffold, such as a biphenyl (tetrazole), highlighted in yellow in Table 1, an imidazole, highlighted in pink, and an alkyl chain, highlighted in purple, following the same rational design principles used in developing the prototype losartan. The different substitutions and modifications on each one of the eight commercially available molecules lead to differences in their water solubility, bioavailability, and affinity for the AT1R, as can be observed by the data presented in Table 1. For instance, the inclusion of an extra aromatic ring and a carboxylic acid, in the case of candesartan, along with the reduction in the length of the alkyl chain, led to a molecule that is significantly less soluble, and with a lower affinity to the AT1R, yet more bioavailable. The absence of the extra phenyl and the imidazole ring in valsartan led to an improvement in its water solubility at the cost of reduced bioavailability but a significantly increased receptor affinity. Analogous observations can be made with the remaining sartans. Nonetheless, although very effective and widely used, the pharmacological profiles of all sartans face challenges due to high lipophilicity and suboptimal bioavailability. To address these issues, ongoing efforts involve exploring new formulations and leveraging advancements in nanotechnology. A recent review by Turek et al. explores the strategy of cocrystallization or formation of coamorphous solids as strategies to address the bioavailability challenges faced by the sartan class.^73^ Within this approach, the low solubility is tackled through noncovalent complexes with several “co-formers” that not only enhance the bioavailability of the sartan used but also broaden the therapeutic application of the resulting formulation, while combining the therapeutic effects of both the sartan and the co-former.

## 2 The Role of Angiotensin II

**Figure 2:**
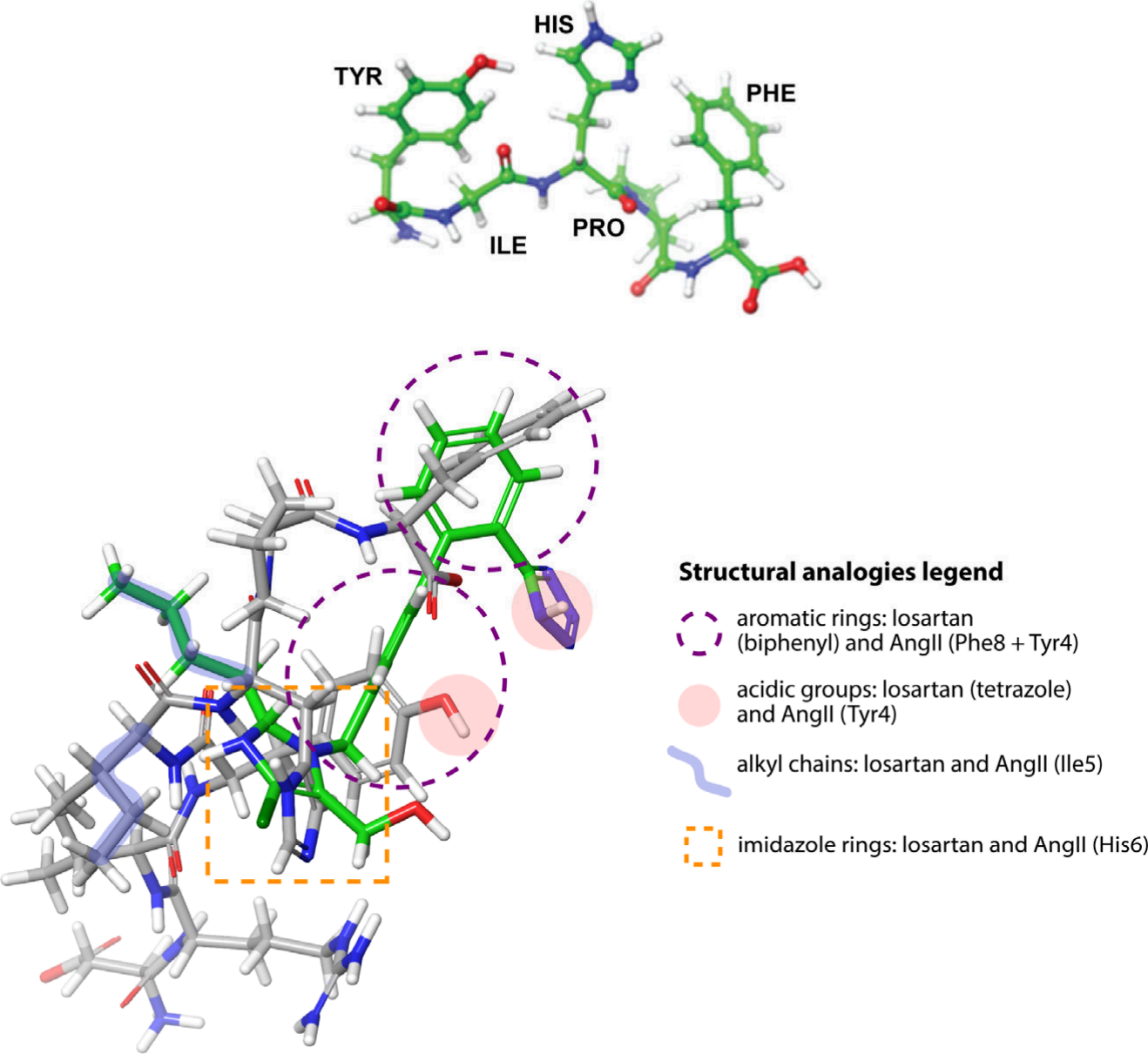
(top) Proposed model of the C-terminus of Angiotensin II. Its major characteristic is the relay system between Tyr4, Hist6, and Phe8 which occurs intramolecularly. (bottom) Superimposition of losartan (green) with AngII (gray). The key analogies between the peptide and the losartan structures are presented in an embedded legend, i.e., the spatial vicinity of the aromatic rings (depicted in purple dashed circles), the acidic groups (in pink circles), the alkyl chains (in blue brush strokes), and the imidazole rings (in orange dashed rectangle).

A lot of effort has been made in order to reveal the bioactive conformation of AngII, due to its major role in regulating RAS function. The initial conformation on which losartan’s design was based—long before high-quality crystal structures were available—was mainly linear.^92−107^ Since then, various conformations have been published, which deviate from one another due to variations in methodology.^103,108−115^ The bioactivity of losartan is based on the mimicry of the C-terminal of AngII, and according to biophysical studies of our group, superposition of losartan on the C-terminal region of Sarmesin (an AngII analogue with the sequence of 1-Sar-4-Me-tyr-angiotensin II) revealed a very good match,^116^ with the butylic group of losartan matching the Ile of AngII and the imidazole matching His (see Figure 8 of ref (116)). A similar model is illustrated in Figure 2, bottom, where losartan is superimposed with the now-resolved EM structure of AngII, bound to AT1R, and their structural analogies are described. Studies of our group as early as 1994 had also proposed a bioactive conformation of Ang II shown in Figure 2, top, based mainly on nuclear magnetic resonance (NMR) studies.^117^ The main characteristic of this model is a suggested Tyr-Ile-His bend and a charge relay system involving the aromatic rings Tyr4-His6-Phe8. In particular, we used the one-dimensional nuclear Overhauser effect (NOE) by irradiating protons on any of the triad of amino acids Tyr-His-Phe, which led us to observe the NOE effect on the other two amino acids. This provided strong evidence for aromatic clustering between the three amino acids. This specific effect was observed with the native hormone Ang II and its [Sar1]AngII superagonist but not with the control pentapeptide [des-1,2,3]AngII. A wide array of biophysical methodologies has demonstrated that the AT1R activation is mediated by a charge-relay system within Ang II, involving the TyrOH-His-Phe carboxylate triad. This system produces a tyrosinate anion, crucial for triggering receptor activation.^118^

**Figure 3:**
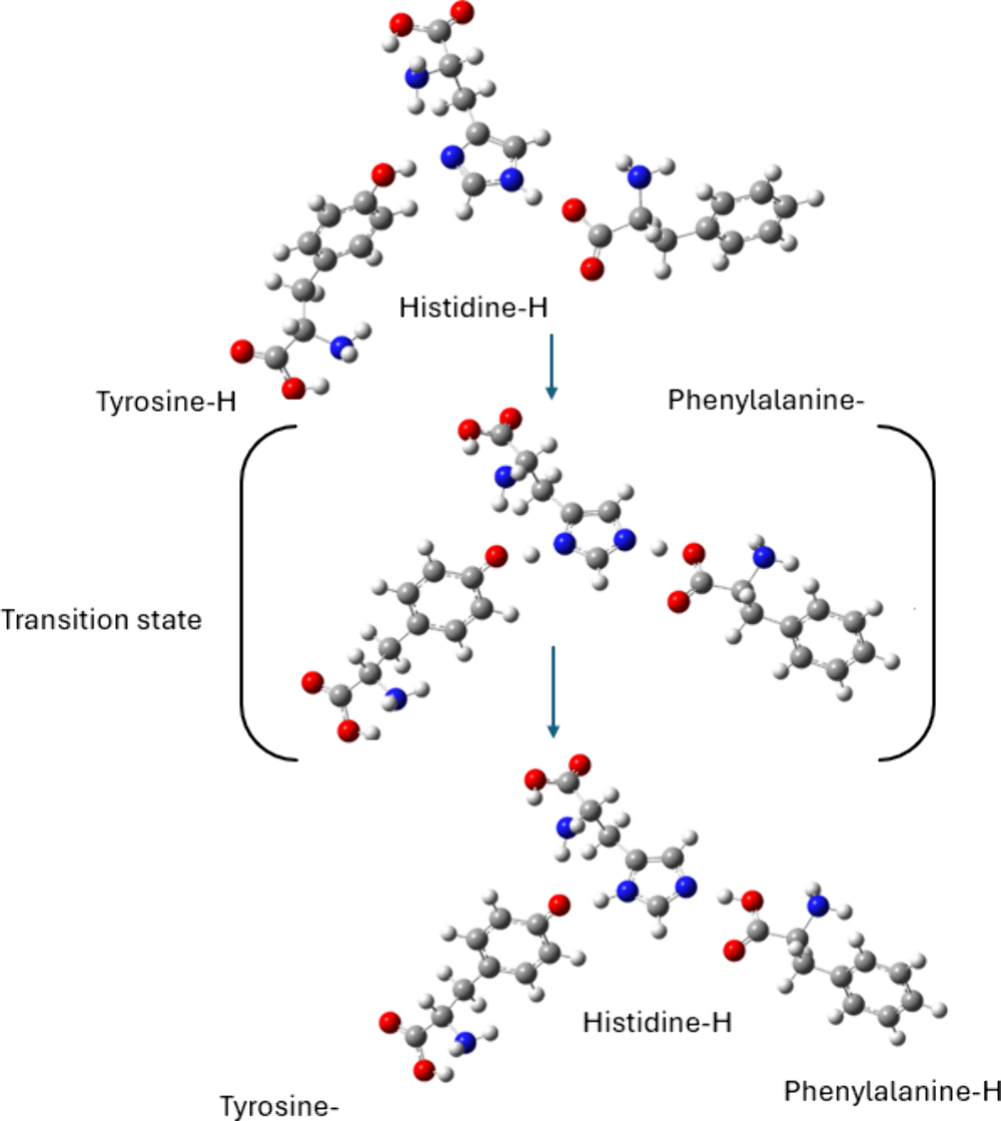
Relay system derived from DFT calculations at the B3LYP/6-311+g(d,p)/PCM(water) level of theory.

A proton relay system among these amino acids was recently confirmed by density functional theory (DFT) calculations performed by our group.^119^ The study examined a possible proton transfer among the AngΙI amino acids, specifically tyrosine, phenylalanine, and histidine. It was found, that while proton transfer in free amino acid heterodimers is unfavorable, it becomes favorable in an amino acid trimer, specifically from tyrosine to histidine with phenylalanine assisting in the process, see Figure 3. Similar findings were found on the Tyr-His-Phe peptide of Angiotensin Converting Enzyme-2 (ACE2) when bound to several sartans.^119^ The findings revealed that the presence of all three amino acids is essential for proton transfer, indicating that they function collectively as chains for this process. Our study underlines the importance of this relay system, given that even outside of the confined space of the receptor’s binding site the amino acids arrange in such a way as to facilitate the proton transfer.

Recently, Kruse et al.^120^ published a crystal structure of AT1R with AngII bound to the receptor’s binding site. The peptide affords several interactions with the binding site of the receptor, mostly including hydrogen bonds, such as Asp263(AT1R)/Arg2(AngII), Asp281(AT1R)/His6(AngII), and Lys199(AT1R)/Phe8(AngII), as well as π–π stacking between Trp84(AT1R) and Pro7(AngII). The crystal structure of AT1R with a model antagonist, released in 2015 by Zhang et al.,^121^ had already provided some insight on the antagonist binding, followed by almost all sartans. In particular, the hydrogen bonds/salt bridges between Arg167(AT1R) and the imidazole ring and the carboxylic groups of sartans are quite decisive for successful sartan binding, whereas they were not present in the case of AngII. The hydrogen bonding with Lys199, however, is a common binding characteristic for both the sartans and AngII. The π–π stacking with Trp84(AT1R) is maintained by the aromatic rings of nearly all sartans, except telmisartan which is bulkier than the rest of the group and is accommodated differently in the binding site.

**Figure 4:**
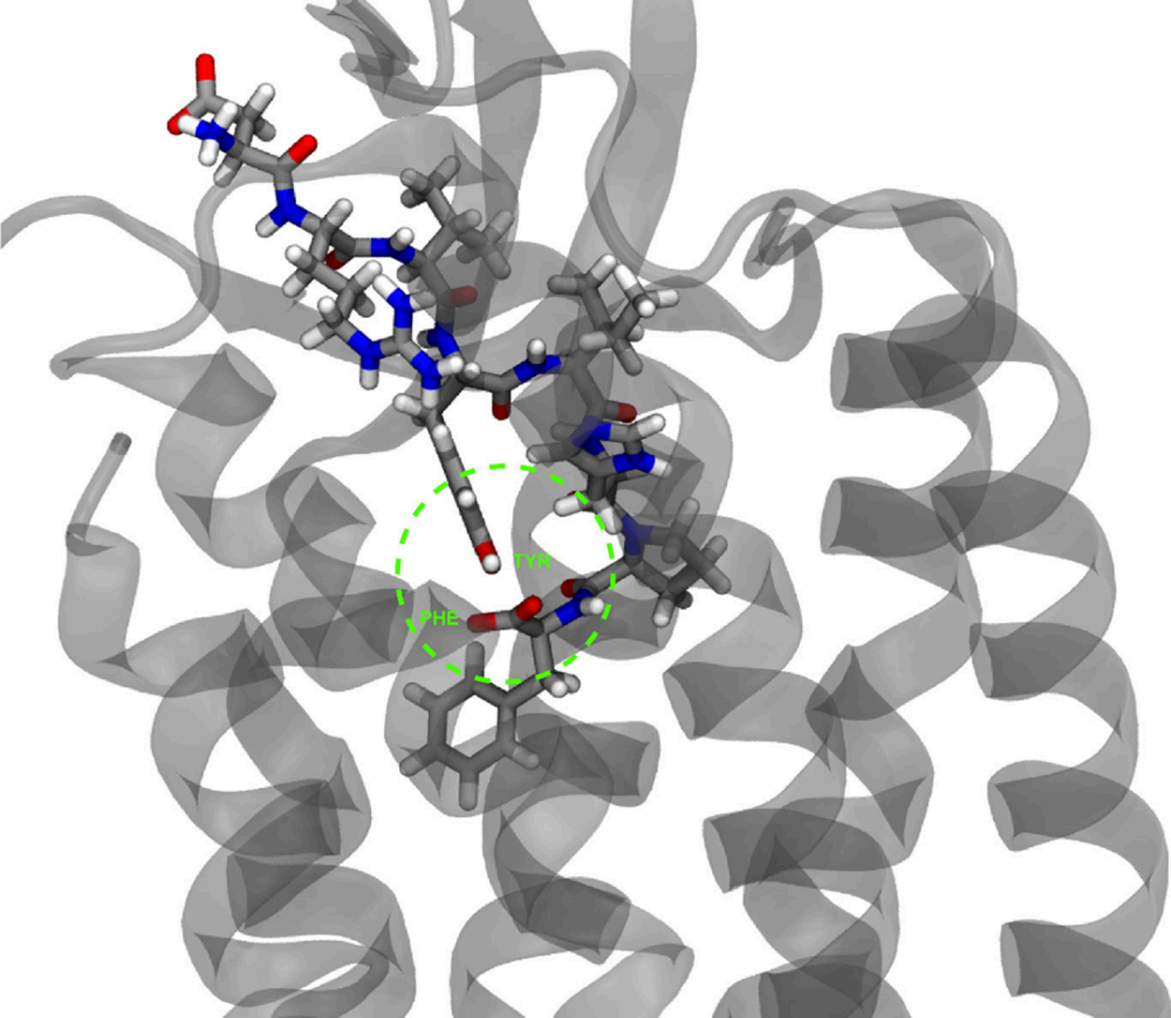
Octapeptide angiotensin II crystallized inside the AT1R.^120^ PDB ID: 6OS0. The conformation adopted by Tyr4 and Phe8 is compatible with that of our proposed proton relay system.

The conformation adopted by the bound oligopeptide in the recent crystal structure is compatible with our proposed proton relay model, with the three amino acids in close vicinity, as presented in Figure 4. Nonetheless, in the confined space of the receptor’s binding site, His does not appear to be important for the proton transfer since the adopted conformation of AngII permits a direct transfer between Tyr and Phe (Figure 4.). Kruse’s work suggested that the presence of the eighth amino acid Phe8 is crucial for invoking the action of the balanced endogenous agonist AngII. They showed that a truncated heptapeptide or a mutated (F8A) AngII analogue failed to generate Gq-dependent inositol phosphate while they could promote β-arrestin-dependent endocytosis,^120^ indicating that absence of Phe8 leads to a β-arrestin biased action. These results underscore the importance of terminal Phe, and further studies are needed to decipher the exact role of this proton relay in AngII’s signaling pathway.

## 3 Angiotensin II Analogues and Novel Formulations

**Figure 5:**
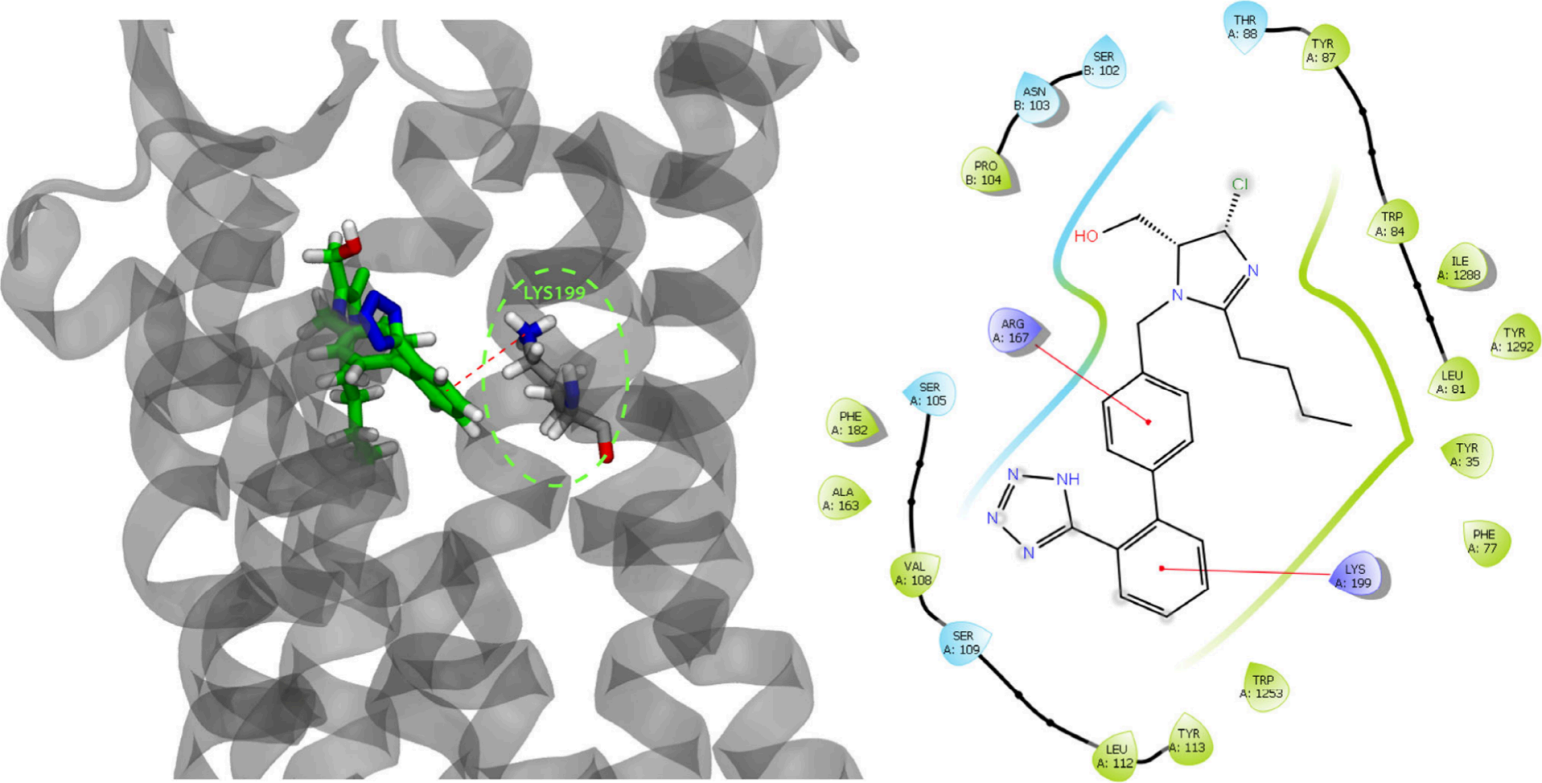
(left) EM structure of losartan inside the AT1R/soluble cytochrome b562 complex. The receptor is illustrated in silver, whereas losartan is in green. PDB ID: 8TH4. Interactions of the π-cation nature are taking place between Lys199 (illustrated in silver, licorice representation) and losartan, also suggested by our docking studies to a receptor’s homology model. (right) Detailed ligand interaction diagram of losartan bound to the EM structure of AT1R.

Due to the important role played by AngII in pathological states, a lot of effort has been made in the past to synthesize novel analogues that antagonize this effect. Before the first release of an X-ray crystal structure of AT1 by Zhang et al. in 2015,^121^ our group has worked with homology models of AT1 receptor. Our docking studies performed in 2003^122^ on an homology model of AT1R are in great agreement with the recent electron microscopy (EM) structure of losartan in the AT1R^123^ (Figure 5), revealing important interactions between the drug molecule and the residue Lys199 of the receptor’s binding site.

**Figure 6:**
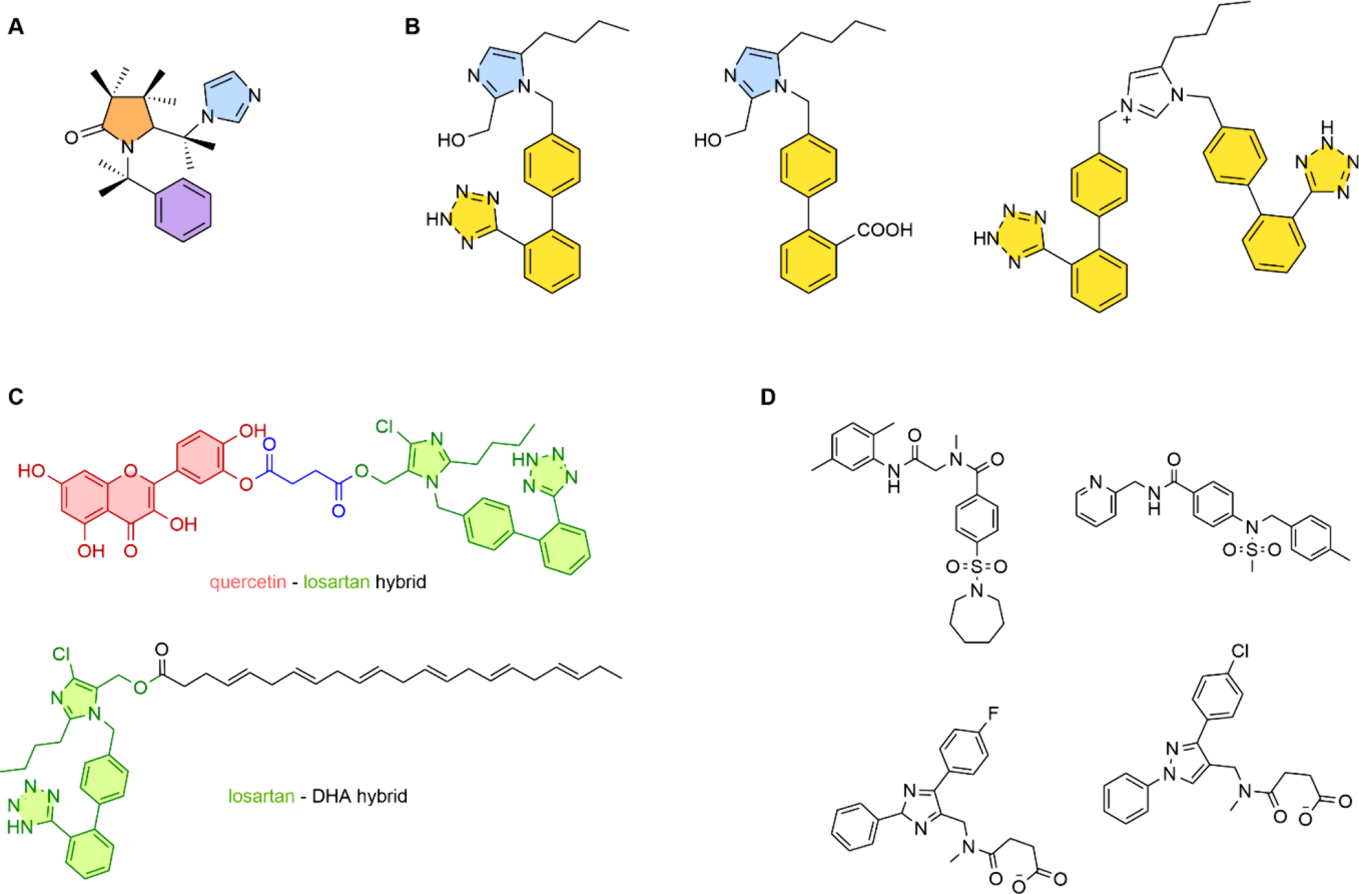
Synthetic AT1R antagonists prepared by our group and collaborators that consist of (A) small, nonpeptide molecules with few synthetic steps, (B) bisartans, (C) losartan-quercetin and losartan-DHA hybrid molecules, and (D) diverse scaffolds resulted from virtual screening of large databases.

Given the known challenges of losartan’s high lipophilicity and low bioavailability, our group has applied many approaches for the synthesis of new analogues that act as antagonists on the AT1R throughout the years. In this review, we briefly mention some of our approaches, presented in Figure 6. One of our approaches was the synthesis of new, nonpeptide small molecules with few synthetic steps, such as the example presented in Figure 6A,^124^ which was designed based on a model of AngII and showed promising biological results. A similar strategy includes the synthesis of new molecules designed to fill a lipophilic cavity that sartans do not accommodate, known as bisartans.,^125−127^ some examples of which are presented in Figure 6B. *In vitro* IC_50_ assays showed promising results with values even similar or superior to that of losartan (ranging from 0.35 nM to 6,.46 μΜ), indicating that the bis-alkylation of the imidazole ring can give rise to a new class of biologically active molecules. We have also proceeded in the design and synthesis of hybrid molecules that exert dual effects,^128,129^ taking advantage of the beneficial effects of natural products such as quercetin^130^ or ω-3 fatty acids such as DHA.^131^ In the case of the quercetin-losartan hybrid, *in vitro* studies showed a promising IC_50_ of 140 nM against the AT1R. The corresponding losartan-quercetin and the losartan-DHA hybrids are presented in Figure 6C. Finally, we have performed virtual screening of large databases, such as the ChEMBL15 in order to discover potential drugs targeting the AT1R with a different scaffold than that of sartans,^132^ examples of which are presented in Figure 6D. The most promising candidates showed IC_50_ values ranging from 257 to 200 nM.

**Figure 7:**
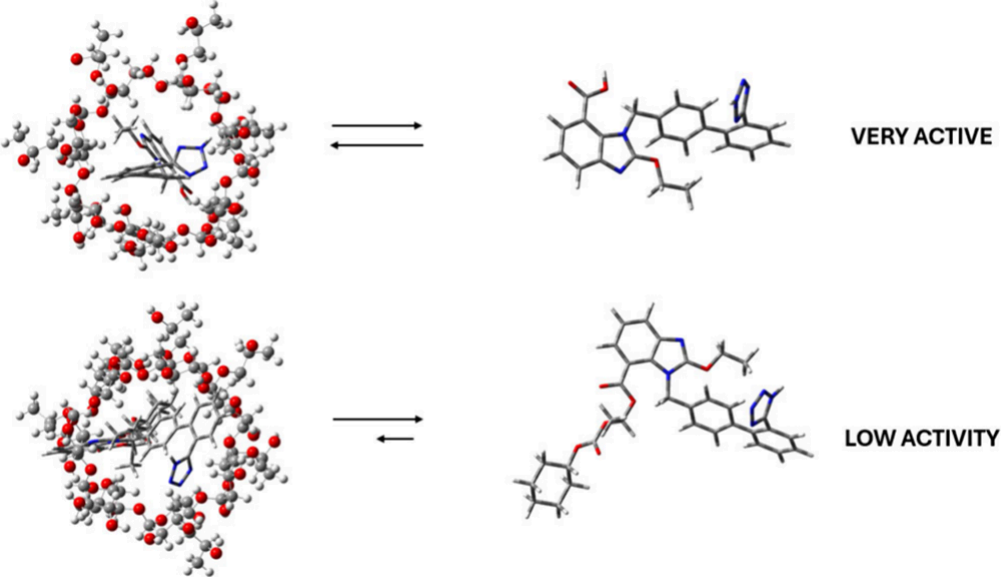
The stronger the interactions between 2-hp-β-CD and the drug molecule, the lower is the latter’s activity as the availability of the drug becomes low and cannot exert its beneficial action. The top, highly active drug molecule is candesartan and the lower, less active is candesartan cilexitil, engulfed in 2-hp-β-CD. For more details on our study refer to ref (136).

**Figure 8:**
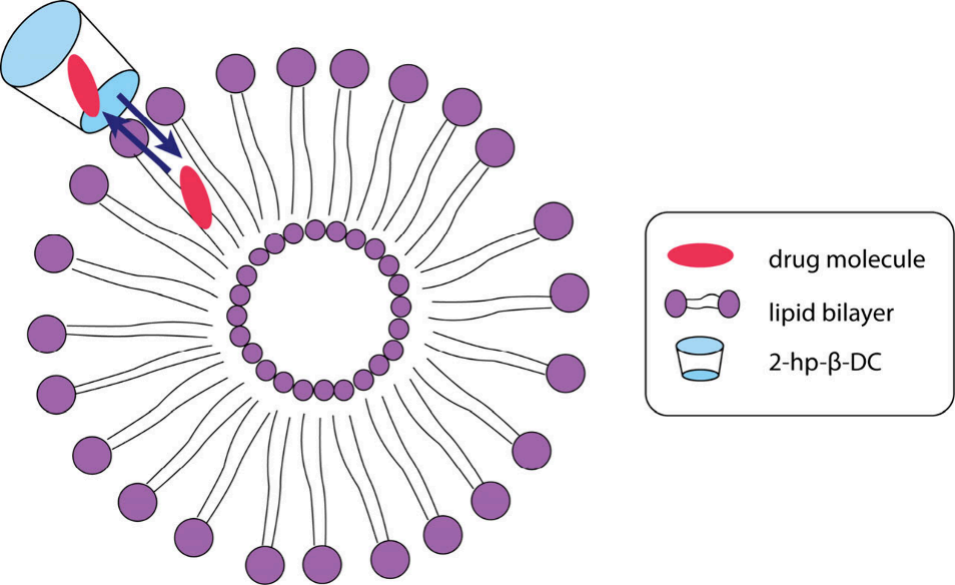
Schematic representation of the drug:2-hp-β-CD complex, its transport to the lipid bilayer, and the subsequent drug release.

Nonetheless, our efforts were not restricted to the design of new molecules, but we also adopted a further approach to improve the bioavailability of existing molecules, that is, the implementation of nanotechnology for their efficient drug delivery. For example, we engulfed losartan,^133^ irbesartan,^134,135^ and candesartan^136^ in a 2-hydroxypropyl-β-cyclodextrin (2-hp-β-CD) in order to study their ability to form stable complexes and assess the strength of their interactions with their host (see Figure 7). Our studies demonstrated that the sartans can be successfully engulfed inside the host, but interactions that are too strong can lead to a low release rate and, as such, to low pharmaceutical activity. A schematic representation of the drug carrier system, its transport to lipid membranes, and the drug release is illustrated in Figure 8.

Starting with the prototype losartan, we recently published a study where its complexation with 2-hp-β-CD was confirmed using a combination of DSC, NMR, and computational studies.^133^ 2D-ROESY NMR experiments showed spatial proximity between losartan’s alkyl chain and aromatic rings when encapsulated, while DFT results showed extensive hydrogen bonding between the tetrazole group of the drug and the hydroxyl groups of the HP-β-CD. Additional water-bridged hydrogen bonds were also present between the spiro moiety of losartan and the cyclodextrin. Molecular dynamics (MD) studies revealed a reversible complexation indicating a possible successful release of the drug molecule, with Δ*G*_MM–PBSA_= −4.8 kcal/mol. An earlier study by De Paula et al.^137^ showed that losartan in its complex formed with 2-hp-β-CD showed an extended duration of action on AT1R, compared to the free form (30 h vs 6 h, respectively). These results provide evidence that this formulation is promising and should be further explored through biological experiments.

Further to our studies with losartan, we have also explored the release of irbesartan by the same 2-hp-β-CD, using an array of biophysical and computational methodologies, such as differential scanning calorimetry (DSC), small-angle X-ray scattering (SAXS), ESI mass spectrometry (ESI-MS), solid state NMR, DFT, and MD. Our computational results showed that a moderately stable complex is formed, leaving the opportunity for the subsequent release of the drug molecule (Δ*G*_MM-PBSA_ = ∼ −11 kcal/mol).^134^ Unlike losartan, irbesartan’s tetrazole was very flexible inside the carrier’s cavity. The biophysical assays indicated release of the irbesartan molecule and strong interaction with model membranes of DPPC bilayers.^135^ Pharmacological evaluation of the irbesartan/2-hp-β-CD complex showed that the drug could successfully inhibit the AT1R, exhibiting similar binding affinities with the uncomplexed drug molecule.^134^ Further studies are currently being conducted by our group, using a combination of one- and two-dimensional liquid-state NMR, in order to account for solvent effects and study the lipid environment in its liquid phase, along with computational studies.

Similarly, we have also explored the interactions of candesartan and its prodrug, candesartan cilexitil with the same cyclodextrin carrier.^138^ The interactions between the drug and the carrier were explored with MD and DFT studies, and as in the case of losartan, the tetrazole moiety of the drug engaged in hydrogen bonding with the hydroxyls of the 2-hp-β-CD. We showed that although candesartan shows weak interactions with the host molecule and can be successfully released, the prodrug candesartan cilexitil exerted stronger interactions with the carrier, leading to reduced activity (Figure 7). Particularly, pharmacological assays showed that complexation of candesartan to 2-hp-β-CD did not alter its binding affinity with AT1R, whereas the prodrug candesartan cilexitil, which exhibited stronger complexation with the cyclodextrin (Δ*G*_MM-PBSA_= ∼ −1.6 kcal/mol vs ∼ −7 kcal/mol for the cilexitil), showed lower binding affinity to the AT1R when in complex with the 2-hp-β-CD. This work highlights that not only a successful complexation but also a successful release rate of the drug molecule should be considered when designing novel drug carrier supramolecules.

**Figure 9:**
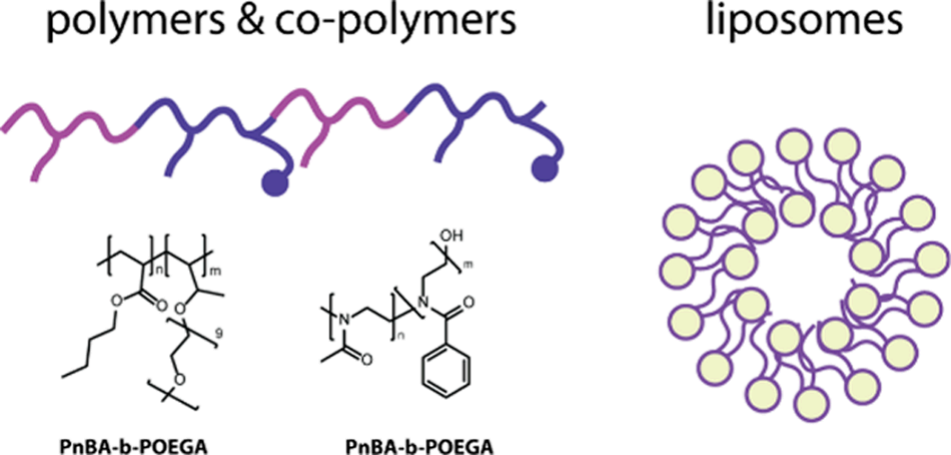
Copolymers and liposomes are used as drug delivery systems.

During our dedicated studies to enhance the bioavailability and delivery of the AT1 blockers, we have used also polymers and copolymers as drug delivery systems^139,140^ (Figure 9). Amphiphilic block copolymers (AmBCs) have been extensively used by the pharmaceutical industry as a novel and sustainable delivery system for the treatment of various diseases, due to their ability to self-assemble and form micelles.^141^ With this architecture, the lipophilic drug molecules can be encapsulated in the lipid core of the resulting micelles, thus enhancing their delivery in the aqueous extracellular environment. We have performed studies using poly[oligo(ethylene glycol) methyl ether acrylate)] (POEGA) copolymers, which are both hydrophilic and biocompatible due to their oligo(ethylene glycol) (OEG) side groups. Our results showed that losartan was successfully encapsulated into the micelle core, while stability studies ensured a stable drug:polymer formulation for up to 23 days.^139^ As a matter of fact, 2D-NOESY experiments demonstrated strong interactions between the biphenyl ring and butyl chain of losartan with the methylene signals of PnBA. As in the case of cyclodextrin, an important factor that needs to be considered in the design of novel nanocarrier formulations is the successful release of the drug molecule when the suitable environment is reached. UV–vis studies showed a slow release of losartan due to strong hydrophobic interactions within the micelle core, suggesting that further studies are needed for the optimization of this novel formulation. An alternative approach studied the encapsulation of losartan into an amphiphilic biocompatible poly(2-methyl-2- oxazoline)-grad-poly(2-phenyl-2-oxazoline) (PMeOxz72-grad-PPhOxz28) gradient copolymer (GC).^140^ This type of copolymer exhibits a gradual variation in its hydrophilic/hydrophobic monomer composition along molecular chains, in contrast to the AmBCs that show an abrupt change. Thus, CGs usually show greater solubility.^142^ Poly(2-oxazolines) are a promising group of polymer therapeutics due to their high biocompatibility and chemical functionality. We envisioned the use of this type of functional material as a promising delivery system for losartan, and its successful encapsulation was confirmed with various biophysical techniques.^140^ Nonetheless, as in the case of AmBCs, the high stability of their complexes with losartan came with the cost of slow drug release that might hinder successful drug delivery. A recent approach used losartan-loaded liposomes as a strategy to deliver the drug molecule across the blood–brain barrier, and its successful delivery was evaluated *in vivo* in hypertensive rats.^143^ Liposomes are a promising alternative strategy for successful drug delivery, since they are nontoxic sphere-shaped bilayer vesicles consisting of phospholipids, which are very well-tolerated and biocompatible. They are easily tailored to target different organs, making them widely used in organ-targeting therapies.^144^ However, despite their advantages, liposomes exhibit several challenges, such as aggregation and drug leakage and are also prone to hydrolysis and oxidation. As an alternative, proliposomes can be used in drug delivery, where liquid liposomes are transformed into solid state and can be rehydrated rapidly in physiological fluid.^145^ Successful oral bioavailabilty was achieved for valsartan encapsulated in a proliposome, in a study by Nekkanti et al.^146^ In this study, the encapsulation efficiency was very high, and *in vitro* studies showed successful drug release. The improved pharmacokinetic profile was confirmed by *in vivo* studies in male Sprague–Dawley rats.

## 4 The Role of the Lipidic Environment

**Figure 10:**
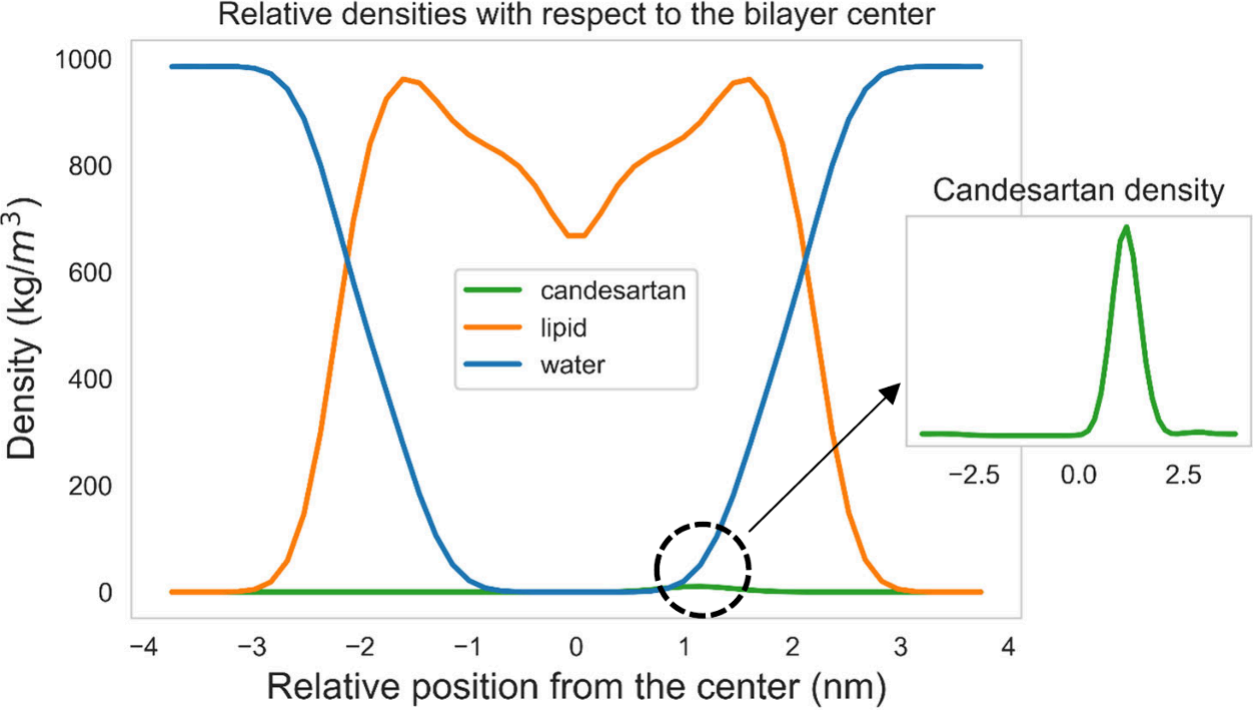
Candesartan’s relative position with respect to the bilayer center. The drug molecule is found to reside at 1 nm off the bilayer center, at the lipid–water interface. Due to the lower concentration of candesartan with respect to the rest of the system’s components, a smaller graph with its density is embedded in the main plot for scaling.

Our efforts have also focused on the interactions of the AT1R antagonists with the lipid bilayers where the receptor is expressed. The AT1R is a transmembrane GPCR and has its binding site located within the lipid bilayer of cell membranes, specifically forming a cavity approximately 2 nm from the center. The involvement of lipid membranes in the drug-binding mechanisms of these receptors has been thoroughly researched by our group^10−16^ and others,^17−20^ with substantial evidence indicating their critical role in drug action. The biophysical studies using solid state NMR spectroscopy, X-ray diffraction, and DSC show that AT1 antagonists can be accommodated between the interface and the lipoidal core of the lipid bilayers. These experimental results have also been confirmed by MD simulations.^14,147−151^ An example of an MD simulation indicating that the relative position of candesartan lies at ∼1 nm of the bilayer center is presented in Figure 10 and thoroughly discussed in ref (152).

**Figure 11:**
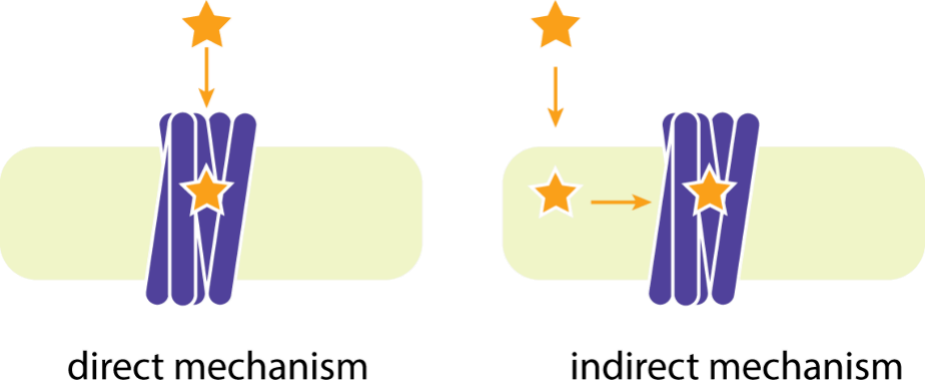
Schematic representation of the direct and indirect mechanisms of drug-receptor binding.

Due to the location of the AT1R’s binding site deep inside the bilayer, we can assume one of the following possible binding mechanisms: a direct one, where the drug approaches its target by 3D diffusion through the aqueous extracellular environment, or an indirect one, where the drug initially penetrates the membrane bilayer followed by 2D diffusion to the transmembrane receptor binding site (Figure 11). There are several examples of both mechanisms for different GPCRs,^153−156^ hence the actual pathway that sartans follow in order to bind to the receptor remains an open scientific question.

**Figure 12:**
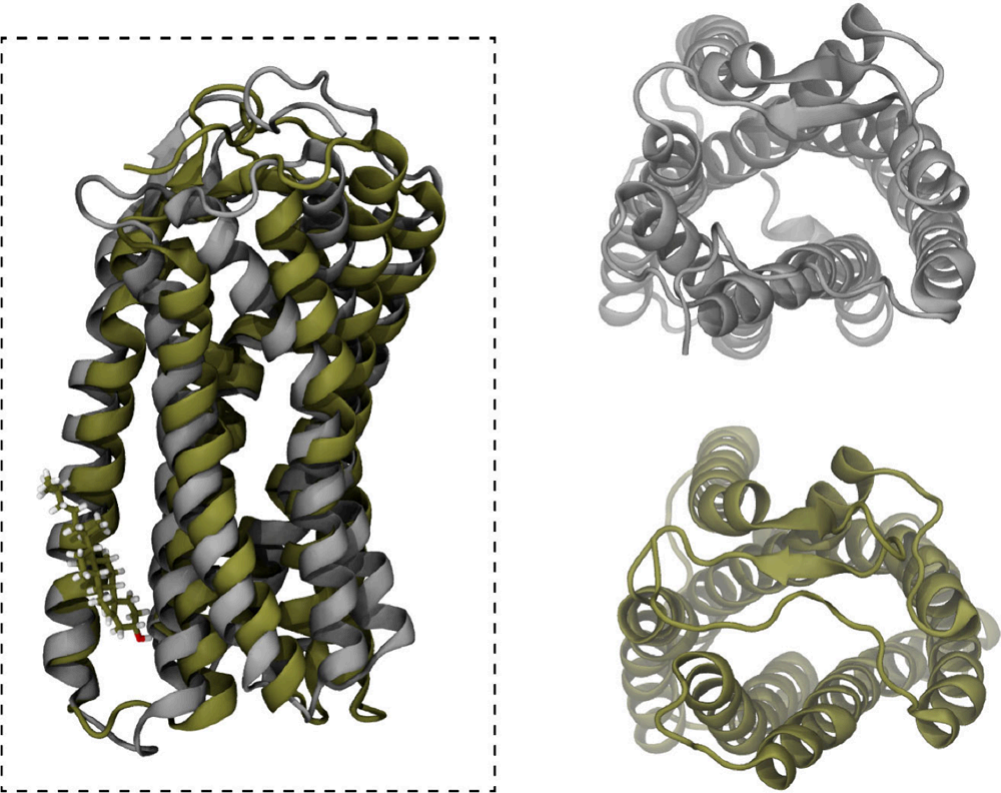
Most prevalent cluster structure of AT1R embedded in pure DPPC (silver) and DPPC:cholesterol (60:40%mol) bilayers. The top views presented on the right clearly indicate that a conformational change invoked by the allosteric binding of cholesterol on AT1R blocks the entrance of the extracellular site to the binding site through the N-terminus. For more details see refs (11 and 12).

Recent studies of our group on the interactions of a highly lipophilic AT1R blocker, candesartan, with the AT1R and the membrane bilayer with^11^ or without^152^ cholesterol, showed that the more realistic membrane model, including the physiological concentration of 40% mol cholesterol, induces conformational changes on the AT1R that may favor the indirect, membrane-diffused binding mechanism. In particular, our studies indicated that a cholesterol molecule strongly binds to an allosteric cavity of the AT1R, namely, a newly discovered Cholesterol Consensus Motif (CCM) on the receptor and induces important conformational changes that affect its N-terminus. The latter gains flexibility compared to the case where pure DPPC membrane models were used and blocks the extracellular entrance of the receptor^157^ (Figure 12). These results indicate that a bilayer-mediated, indirect mode of binding could be a more possible mechanism for candesartan-AT1R binding. This result is quite promising, as confirmation of the indirect mechanism could transform the high lipophilicity, typically a drawback of most sartans, into a significant advantage. A strategy focusing on novel formulations with drug carrier systems, such as cyclodextrins, polymeric micelles, or liposomes, could help overcome the problematic solubility of sartans in physiological fluids while leveraging their high lipophilicity near cellular membranes.

## 5 Conclusions

Hypertension stands as a major contributor to premature mortality globally, and this issue is anticipated to be exacerbated in the forthcoming years. Effective management of hypertension is crucial for reducing this burden; thus, research on advancing hypertension therapy and suggesting novel pharmaceutical interventions plays a vital role. Among these interventions, AT1R antagonists, with their prototype losartan, have shown promise due to their targeted action on RAS. Our research highlights the multifaceted role of losartan and its analogues in mitigating hypertension and related complications. Losartan’s efficacy in blocking the detrimental effects of AngII has been well-documented, providing benefits in conditions such as diabetic nephropathy, heart failure, stroke prevention, and potentially even COVID-19. However, challenges such as high lipophilicity and suboptimal bioavailability necessitate ongoing efforts to enhance its pharmacological profile. Advancements in nanotechnology and novel formulations are paving the way for improved drug delivery systems. Our studies on the encapsulation of several sartans in safe and biocompatible nanocarriers such as 2-hp-β-CD demonstrate promising results. These formulations not only ensure stable complexation but also facilitate effective drug release, which is crucial for maintaining therapeutic efficacy. In addition, our group’s research has focused onto hybrid molecules, such as losartan-quercetin and losartan-DHA hybrids, which aim to combine the antihypertensive effects of sartans with the additional therapeutic benefits of natural products and omega-3 fatty acids, respectively. These hybrids have shown promising biological results, indicating the potential for enhanced therapeutic efficacy. Further, the design of bisartans, which accommodate a lipophilic cavity not filled by traditional sartans, has led to molecules with improved binding affinities and biological activities. *In vitro* assays have demonstrated that these bisartans exhibit similar or superior IC_50_ values compared to losartan, indicating their potential as powerful antihypertensive agents. Moreover, virtual screening of large databases has identified novel scaffolds targeting AT1R, expanding the arsenal of potential drugs beyond the traditional sartan framework. The lipidic environment’s influence on AT1R conformation and drug binding underscores the complexity of drug-receptor interactions. Our findings suggest that the lipid bilayer plays a critical role in modulating receptor dynamics and drug binding. This understanding is crucial for designing more effective antihypertensive therapies that leverage the lipidic milieu of cellular membranes. Overall, the continuous refinement of AT1R antagonists, informed by a deep understanding of their molecular interactions and pharmacodynamics, holds promise for more effective hypertension management. Furthermore, in this review, we highlighted the significance of computational simulations as a valuable tool for comprehending and advancing new pharmaceuticals. Future research should focus on optimizing drug delivery systems, exploring new molecular analogues, and further elucidating the role of lipid membranes in drug action. By addressing these areas, we can enhance the therapeutic potential of AT1R antagonists and contribute to better cardiovascular health outcomes globally.
